# Effect of Instant Controlled Pressure-Drop (DIC), Cooking and Germination on Non-Nutritional Factors of Common Vetch (*Vicia sativa* spp.)

**DOI:** 10.3390/molecules25010151

**Published:** 2019-12-30

**Authors:** Angel I. Hernandez-Aguirre, Carmen Téllez-Pérez, Alejandra San Martín-Azócar, Anaberta Cardador-Martínez

**Affiliations:** Departamento de Bioingenierias, Tecnologico de Monterrey, Monterrey 76158, Mexico; ibq.anivhdez@gmail.com (A.I.H.-A.); ctellezpe@tec.mx (C.T.-P.); alsmartin@tec.mx (A.S.M.-A.)

**Keywords:** non-nutritional factors (NNFs), vetches (*Vicia sativa* spp.), instant controlled pressure drop (DIC), cooking, germination, total phenolic compounds, tannins, oligosaccharides, phytic acid

## Abstract

Legumes are widely consumed by humans, being an important source of nutrients; however, they contain non-nutritional factors (NNFs), such as phytic acid (IP_6_), raffinose, stachyose, total phenolic compounds, condensed tannins, and flavonoids, that have negative effects on human health. Although vetches (*Vicia sativa*) are widely cultivated, they are not intended for human feeding due to their contents of NNF. Usually, the NNF are removed by cooking or germinating; however, germination is a process that requires extended time, and cooking may compromise the viability of some nutrients. To promote vetches for human consumption, the effect of the Instant Controlled Pressure Drop (DIC) process was studied as an alternative to cooking and germinating to decrease NNF contents. Results showed that compared to raw vetches, DIC treatment reduced total phenolic compounds (48%), condensed tannins (28%), flavonoids (65%), IP6 (92%), raffinose (77%), and stachyose (92%). These results are very similar to the ones achieved by traditional ways of removing NNF.

## 1. Introduction

According to the Food and Agricultural Organization (FAO), pulses are dry seeds of plants belonging to the *Leguminosae* family. Within the most common pulses, stand out beans, broad beans, peas, chickpeas, cowpeas, pigeon peas, lentils, Bambara beans, lupins, vetches, and other minor legumes [1]. It is known that pulses represent an important source of proteins and nutrients necessary for human feeding [2]. Moreover, numerous studies suggest that consumption of legumes may have potential health benefits as reducing the risk of cardiovascular diseases [3], cancer [4], diabetes [5], hypertension [6], among others. In México, according to FAO, the most consumed legume are common beans (*Phaseolus vulgaris*); nevertheless, they present a low crop yield [7]. On the other hand, *Vicia sativa spp.* (common vetch) is not commonly used for human feeding, but is widely cultivated as soil improvement and as feed for livestock due to their high content of proteins and other nutrients [8]. Moreover, by comparing to common beans, vetches have a better crop yield [7]. However, the use of pulses such as vetches is reduced due to the presence of compounds known as Non-nutritional factors (NNF). NNF are defined as compounds that reduce the nutrient utilization and/or food intake of plants or plant products used as human foods or animal feeds [9]. Among the NNF identified on pulses, phytic acid, oligosaccharides, and phenolic compounds are the most commonly linked to the most undesirable physiological reactions such as flatulence, inhibition of enzymes, or vitamin absorption and low digestibility [8]. Phytic acid (IP_6_) is one of the most common heat resistant NNF in plants. IP_6_ chelates micronutrients and reduces their bioavailability for monogastric animals, including humans, because of the lack of phytase enzyme in their digestive tract [10]. Oligosaccharides, such as raffinose and stachyose, are well known to cause flatulence in mammals that have no α-galactosidase [11]. These saccharides contain one, two or three galactose units joined to α-1-6 galactosidic linkages. The lack of α-galactosidase leads to the production of flatus gases (H_2_, CO_2_ and small amounts of CH_4_), diarrhea, and discomfort [12]. Phenols bind to positively charged proteins, amino acids, and/or multivalent cations or minerals such as Fe, Ca, and Zn in foods, and decrease their digestibility [13].

However, NNF have also shown health benefits as the improving of essential minerals bioavailability [14], the reduction of the blood glucose and insulin responses to starchy foods and/or the plasma cholesterol and triglycerides [15], the prevention of kidney stone formation, caries, atherosclerosis, and coronary heart disease as well as against of cancer [16]. According to Muzquiz, et al. [17], exposure time, biochemistry, concentration, and interaction with other dietary components, may influence the balance between beneficial or deleterious effects of NNF consumption. Therefore, a relevant challenge is to determine the adequate processing conditions to preserve the adequate amount of NNF in legumes to make the most of the positive effects in human health and to reduce as far the negative effects [18,19,20].

To reduce the NNF of pulses, various treatments such as soaking, fermentation, germination, washing, heating, among others, have been applied [21,22,23,24,25,26,27]. Among all these treatments, heat is the most used, with cooking times between 20 to 120 min. However, the final nutritional value of legumes can be damaged in the function of the type and intensity of heating [28,29,30]. Then the use of HTST (High Temperature/Short Time) treatments becomes promising to reduce the non-nutritional factors [11].

**Figure 1:**
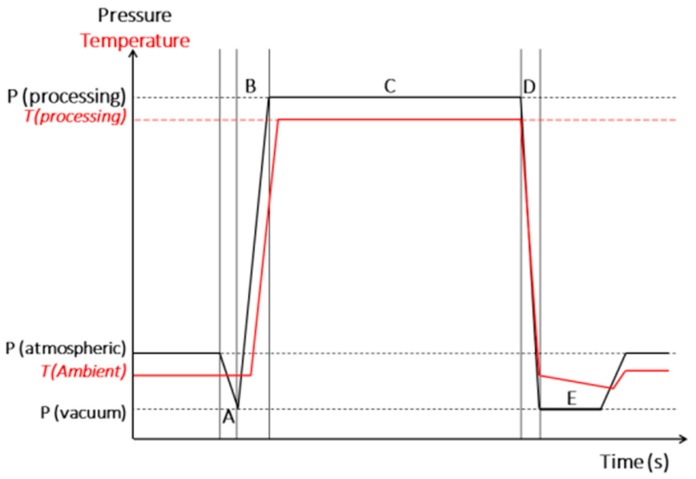
Schematic time-pressure profiles of a DIC processing cycle.

The instant controlled pressure drop technique, better-known by its French acronym Détente Instantanée Contrôlée (DIC) is an HTST treatment. It consists of subjecting a product to a high pressure saturated dry steam (almost between 100 and 1000 kPa according to the product and the objectives) for a short period (seconds), followed by an abrupt pressure drop towards a vacuum (about 5 kPa). This thermo-hydro-mechanical process induces instant auto vaporization of a quantity of the product water, which provokes a controlled expansion and an immediate cooling of treated products, which stops thermal degradation [31]. The effect of DIC treatment has been previously evaluated by Haddad and Allaf [32] and Pedrosa et al. [11] on several NNF of soybean, lupin, lentil, chickpea, and roasted peanut. Their results showed an important reductions of the NNF. Figure 1 shows the schematic time-pressure profiles of a DIC processing cycle.

On the other hand, germination has also been applied to decrease the NNF of pulses [33,34,35,36]. Germination starts when the dry seed begins to take up water and is completed when the embryonic axis elongates [37]. However, the effect of germination depends on the type of legume and the conditions and duration of the germination process [35,36,38].

Therefore, this work aims to compare the impact of the instant controlled pressure drop (DIC) technique to cooking, and germination, on the reduction of NNF of vetches, to lay the basis for possible future use as human food.

## 2. Results and Discussion

### 2.1. Chemical Proximate Analysis

Table 1 shows the average contents of moisture, ashes, crude fiber, total nitrogen, and ethereal extract of non-treated flours. These results are similar compared to the study of bitter vetch seeds performed by Sadeghi et al. [25]; the slight differences can be attributed to the growing conditions and postharvest handling. Proximate analysis performed on others legume flours (chickpea, pea, common bean and lentils), showed that by comparing these legumes to vetches, the latter presented higher contents of crude fiber (5.69 to 10.4 g/100 g dry matter), ashes (3.12 to 4 g/100 g dry matter) and lipids content (2.34 to 6.73 g/100 g dry matter), and lower protein content (18.5 to 23.7 g/100 g dry matter) [39].

### 2.2. Effect of Instant Controlled Pressure Drop, Cooking and Germination on Non-Nutritional Factors of Vetches

Total phenolics content (TPC), condensed tannins content (CT), total flavonoids content (TFC), phytic acid content (IP_6_), raffinose content, and stachyose content of DIC, cooked, germinated and raw vetches are shown on Table 2. Raw vetch flour have shown total phenolics compounds of 191.4 mg eq. of gallic acid/g dry basis; condensed tannins of 12.31 mg eq. of catechin/g dry basis; flavonoids of 16.55 mg eq. of rutin/g dry basis; phytic acid (expressed as mg/g dry basis) of 13.5; raffinose (expressed as mg/g dry basis) of 7.86; and stachyose of 24.67 mg/g dry basis.

#### 2.2.1. Effect of Instant Controlled Pressure Drop Treatment on NNF of Vetches

To better evaluate the effect of DIC treatment on the total phenolic compounds, condensed tannins, flavonoids, phytic acid, raffinose, and stachyose of vetch flours, obtained results were expressed as percentage respect to NNF of non-treated vetch flour (Table 3).

The most significant reductions for total phenolics (48%), condensed tannins (28%), phytic acid (92%), raffinose (77%), and stachyose (92%) are observed at 0.41 MPa. In the case of flavonoids, the most significant reduction (67.43%) was found between 0.3 to 0.33 MPa and 195 s. For raffinose, the highest reduction (77.42%) was obtained under 0.41 MPa and 78 s.

**Figure 2:**
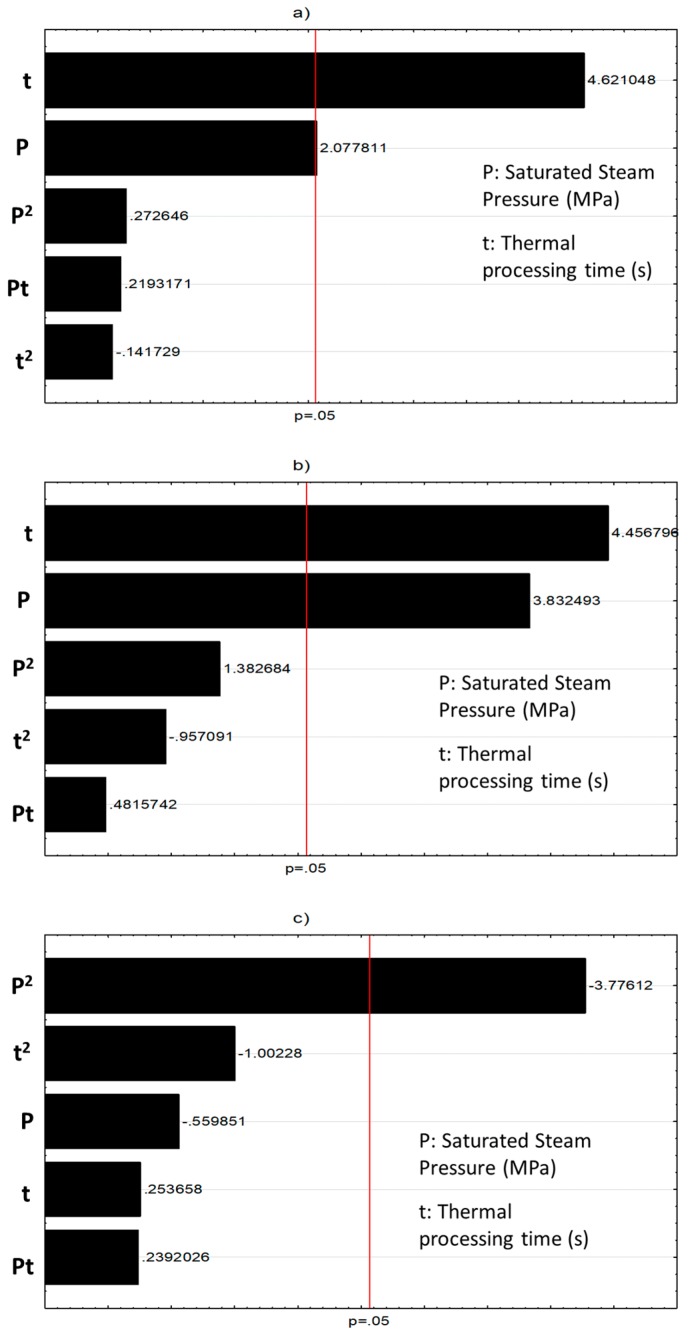

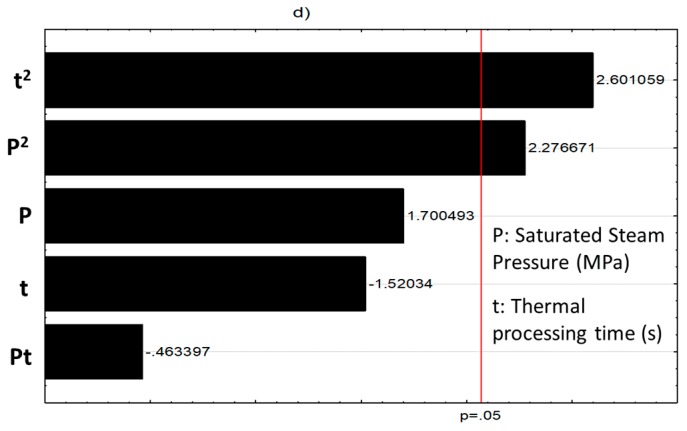
Pareto charts of significative effects on DIC treatments. (**a**) Total phenolics reduction, (**b**) Condensed tannins reduction, (**c**) IP_6_ reduction, (**d**) Stachyose reduction.

**Figure 3:**
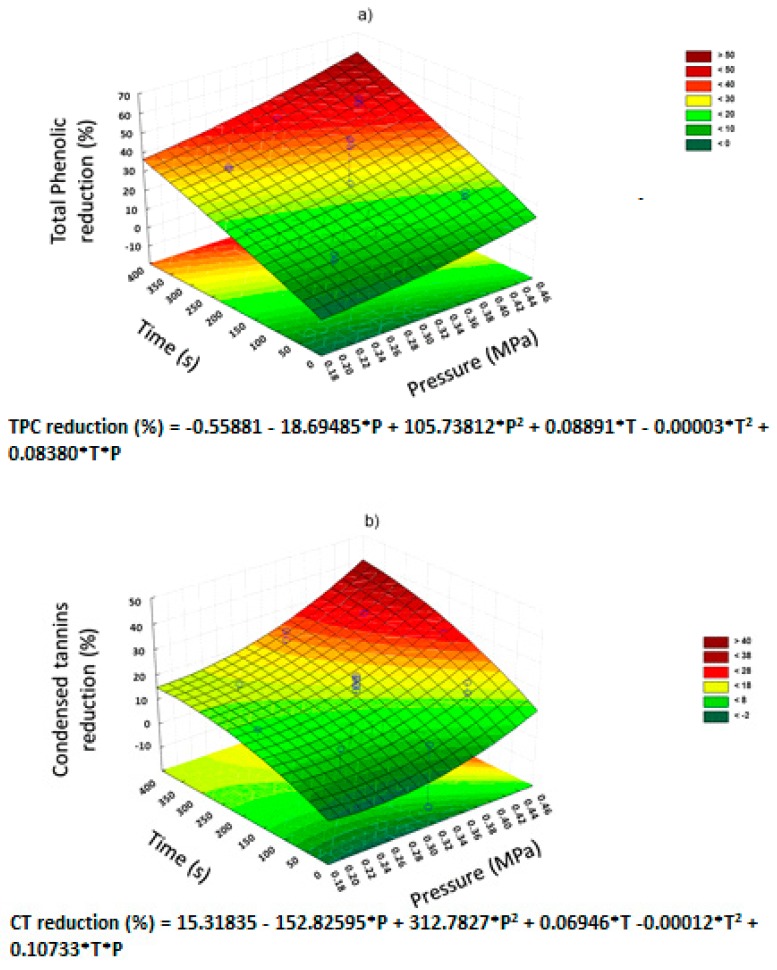

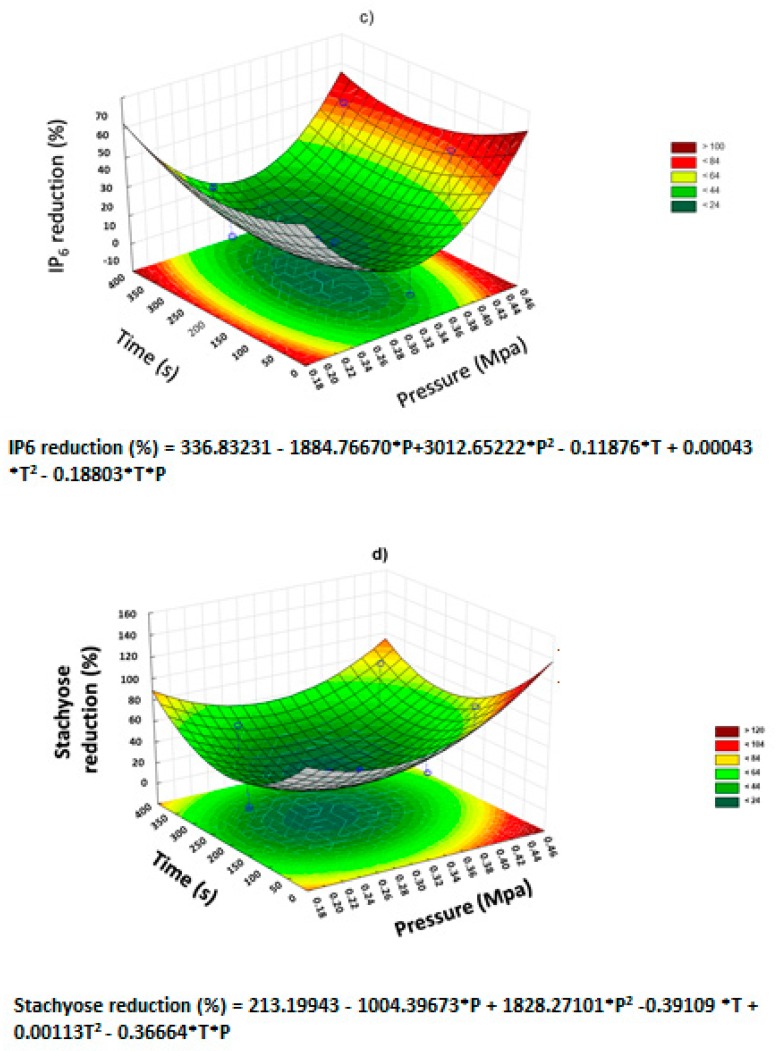
Surface response plot of NNFs reduction by DIC treatment. (**a**) Total phenolics, (**b**) Condensed tannins, (**c**) IP_6_, (**d**) Stachyose. P = pressure; T = Time.

Figure 2 shows the Pareto charts of the significative effects of DIC treatments, and Figure 3 shows the surface response graphs of NNFs reduction caused by DIC treatment. Pareto charts (Figure 2), shows the impact of DIC operating parameters (saturated steam pressure and treatment time) on the reduction percentage of NNFs. In the case of total phenolics (Figure 2a) and condensed tannins (Figure 2b), it can be observed that both pressure and time have a significant effect on the reduction of these NNFs. In fact, the higher the steam pressure and treatment time, the higher the reduction of total phenolics and condensed tannins (Figure 3a,b, respectively). Figure 2c illustrates the significant quadratic effect of saturated steam pressure on IP_6_. By exploring the response surface graph (Figure 3c), it can be observed that under the higher and lower values of saturated steam pressure, a significant IP_6_ reduction can be obtained. With respect to stachyose, Figure 2d shows that both the quadratic effect of saturated steam pressure and the quadratic effect of treatment time have a significant impact on the reduction of stachyose. Moreover, Figure 3d shows that an important reduction of stachyose can be achieved under high values of the saturated steam pressure and low values of treatment time. In the case of flavonoids and raffinose reduction, it can be observed that under the selected operating conditions of steam pressure and time, neither P nor t presents a significant effect on both response variables. Then, the best DIC treatment condition to reduce the most NNF of vetches was DIC 5 (0.41 MPa and 312 s).

In the study of Pedrosa et al. [11], the optimal DIC conditions to achieve a global reduction of NNFs of different legumes were 0.6 MPa and 60 s. In the specific case of oligosaccharides, DIC reduced the raffinose concentration for all legumes in 5%, 7%, 9%, 18%, and 24% for soybeans, lupins, roasted peanuts, chickpea, and lentils respectively. Not the same behavior for stachyose, where for roasted peanuts, lupin, and chickpea DIC allowed a concentration reduction of 11%, 13%, and 14% respectively; however, for lentils and soybean, the concentration of this sugar was increased on 12% and 3% respectively. By comparing to our results, it can be observed that there is a considerable difference between the oligosaccharides reduction of those legumes and vetches (77% for raffinose and 92% for stachyose). This difference could be linked to the particle size of the raw material and the DIC treatment time (1 min vs. 5 min). Whole meals of legumes were submitted to DIC treatment in the study previously described, however, in this work we used vetches flour. Besides, the longer treatment time could allow the increase of sugar leaching on the steam.

According to Haddad and Allaf [32], their results showed that their applied operating parameters, DIC treatment could achieve different reduction ratio of NNF, being remarked that under high pressure treatment (0.70 MPa) and short time (60 s) the NNF of soybeans and lupins seeds (*L. albus* and *L. mutabilis)* could be reduced to 94%, 16%, and 19% respectively

Furthermore, respect to IP_6_ content, the study of Pedrosa et al. [11] showed that DIC treatment allowed its reduction for all legumes: lupin (91%), lentil (51%), chickpea (45%), soybean, and roasted peanut (34%). By comparing to our results, it could be observed that according to the DIC treatment conditions, IP_6_ reduction could vary from 12% to 92%. IP_6_ reduction of vetches could be linked to the thermal degradation of these molecules, the formation of insoluble complexes and the changes in their chemical reactivity during the DIC treatment [40].

On the other hand, according to Yağcı and Evci [41], the DIC process increased the total phenolic content of chickpea varying according to the treatment conditions. As higher the processing pressure and treatment time, the polyphenols content was increased, achieving the highest content (twofold respect to the control) at 0.5 MPa and 10 min. By comparing to our results, it could be observed an opposite behavior; this difference could be linked to the intrinsic chemical composition of each legume and their initial moisture content (30% in chickpea and 15% in vetches). Depending on the achieved temperature during DIC treatment, and the initial water content of food polymers, the rate of vapor generated by auto vaporization during the DIC treatment starts acting as a swelling gas on the concerned polymer. Then according to the initial moisture content, DIC treatment can generate structural expansion, which increases the extraction of biomolecules, such as polyphenols and tannins [42]. Moreover, in this study, phenols reduction could be also linked to its decarboxylation during heating of DIC treatment. The reduction percentage of phenolics compounds achieved by DIC treatments, are accordingly to those reported by Xu and Chang [29], where not only a reduction of phenolic compounds was reported, but an increase of antioxidant activity in steamed legumes. Since DIC is a HTST treatment that consist in high pressure steam, it could be that vetches show improved antioxidant activity after DIC treatment.

#### 2.2.2. Effect of Cooking on NNF of Vetches

The effect of cooking on the NNF of vetches respect to RM is shown in Table 4. The cooking time of 45 min showed the lowest raffinose (91.3%), stachyose (81.7%), and phytates (87.78%) reductions. Phytates reduction showed no significative difference in times above 45 min. The reduction of phytic acid is probably due to hydrolysis during cooking and also caused by the formation of insoluble complexes [43].

Raffinose showed higher reduction after 120 min of cooking time (96%), while stachyose showed their highest reduction (95%) with no significative differences among 60–90 min. Ibrahim et al. [22], reported reductions around 100% of raffinose and stachyose of chickpea soaked in 0.03% sodium bicarbonate solution (16 h) followed by two heating methods: cooking at 100 °C for 45 min and pressure cooked (1 kg/cm^2^) at 120 °C during 20 min. Since oligosaccharides are relatively heated stable, their reduction is mainly attributed to their diffusion into the water during soaking and cooking [44,45]. Moreover, according to the solubility and diffusion rate of each oligosaccharide, sugar losses could be enhanced by increasing the soaking and cooking time and employing different soaking media [45].

The highest flavonoids reduction was found at 60 min of cooking (51.17%), with no significant difference among other treatments with high flavonoids reduction. In the case of condensed tannins, no significant differences were found among the four different cooking treatments, showing reductions from 13% up to 20%. Total phenolics reduction showed no significant differences between C45 and C60; moreover, after 60 min of cooking the highest reduction of total phenolics was achieved (39.52%). The reductions are in agreement with those reported for colored beans and chickpeas [30]. Khandelwal et al. [23] also reported reductions of total polyphenol and tannins of four legumes cooked until softness (121 °C, 15 psi): lentil (41% and 36%), green gram (41% and 45%), Bengal gram (35% and 28%), and red gram (45% and 34%), respectively. Similar results are reported by Xu and Chang [29], which showed that boiling legumes reduced phenolic compounds up to 50%, however, for steamed legumes, the reductions were lower (up to 28%), but they also reported an increase of the antioxidant activity due to polyphenols. Then, the reduction of polyphenols and tannins could be attributed to both, its diffusion to the water during soaking and cooking [46], and the destruction or transformation of its chemical structures during heat treatment [47].

#### 2.2.3. Effect of Germination on NNF of Vetches

The effect of germination on the NNF of vetches respect to RM is shown in Table 5. As can be observed, germination boosted the reduction of raffinose (96%), stachyose (95%) and IP_6_ (95%), however, not the same behavior was found for total phenolics, flavonoids, and condensed tannins which increased its concentration on 28%, 27%, and 4% respectively.

Respect to oligosaccharides, similar results were founding on cowpeas, where 85% and 95% stachyose reduction occurred after germination at 35 °C for 48 h and 72 h respectively; moreover, after 48 h of germination at 30 °C stachyose was disappeared [48]. According to Martín-Cabrejas, et al. [49] germination increase the activity of the enzyme α-galactosidase, which hydrolyzes the α-1-6-galactosidic linkages, thereby causing an efficient reduction of the α-galactosides content.

On the other hand, phytic acid reduction on germinated vetches could be linked to an increase in phytase activities. In fact, this enzyme makes the phytates soluble to support seedling growth [50]. Similar results were found on chickpea after 48 h of germination, where phytic acid content was reduced on 59% [51]. Our results are in agreement with those reported by Mohammed, et al. [52], where the germination significantly decreased the content of phytic acid of lupin (*Lupinus albus*) due to phytase activity, that increases over time.

Respect to total phenolics, flavonoids, and condensed tannins content, most studies found that germinated seeds presented a higher content of these molecules than raw seed. For example, Dicko, et al. [53] reported an increase of total phenolics and flavonoids in different sorghum varieties, due to the activity of enzymes stimulated during germination. Xu, et al. [54] found a significant increase in the total soluble phenolic compounds (free + soluble bound phenolics) of germinated chickpea and yellow pea. Lin and Lai [55] reported that after long-term germination, the contents of bioactive compounds (total phenolics and flavonoids) significantly increased in black soybeans. According to López-Martínez et al. [37], glucose is the original precursor for the synthesis of phenolic compounds, and several crucial molecular signaling pathways, including hydrolyzable tannin pathway. Duenas, et al. [56] also reported an increase of total phenolics and flavonoids, on the germination of lupin seeds, however, the authors reported an increase of 84% of phenolics compounds, after 9 days of germination. Then in function of the kind of legume and the germination process (seeds water content, temperature, lightness or darkness, days of germination, oxygen concentration, among others) the final content of polyphenols on germinated legumes could varied significantly. Although germination significantly reduce IP_6_, raffinose, and stachyose within our germination time, these reductions can be improved if germination is extended to 9 days or more. This also could lead to higher increase of total phenolics, flavonoids, and tannins, that could improve some beneficial aspects to human health, such as antioxidant activity.

### 2.3. Principal Component Analysis

**Figure 4:**
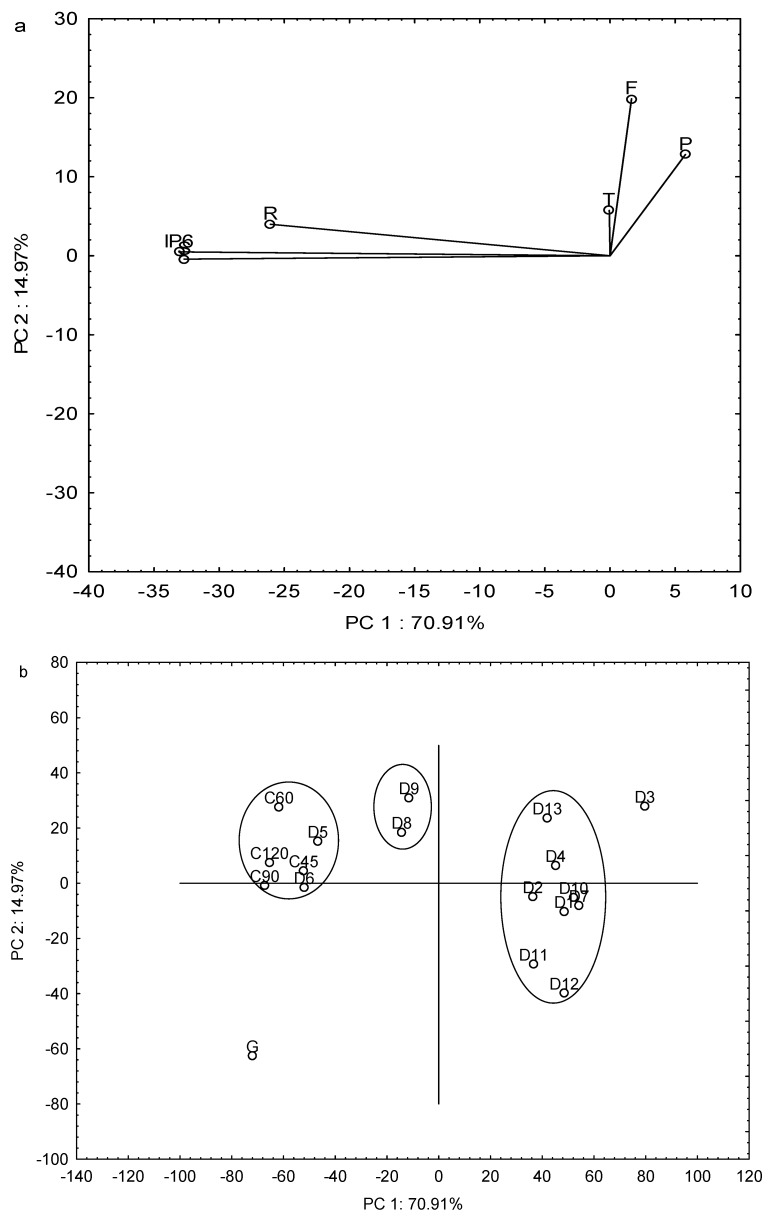
Principal component analysis. (**a**) Projection of the variables on the factor-plane, (**b**) Projection of the cases on the factor-plane. P = Total phenolics, T = condensed tannins, F = flavonoids, R = raffinose, S = stachyose, G = germination, C = cooking, D = DIC treatments.

To reduce the number of response variables, a principal component analysis (PCA) was done. Six components where obtained, where principal component 1 (PC1) and principal component 2 (PC2) described 85% of the variability. Figure 4 shows the classification of the three different treatments (cooking, germination, and DIC) applied to vetches in function to PC1 and PC2. Figure 4a shows that IP_6_, stachyose, and raffinose reduction have a higher contribution to PC1 construction, while total phenolics, condensed tannins, and flavonoids have a higher contribution to PC2. The resultant biplot (Figure 4b) shows three different groups respect to PC1. The first is formed by cooking treatments (C45, C60, C90, and C120) and DIC treatments (D5 and D6) characterized by the highest IP_6_, stachyose, and raffinose reduction. Although D5 and D6 were performed at the same pressure (0.41 MPa), they differ in process time (312 and 78 s, respectively). A second group is only formed by D8 and D9, which is characterized by sharing the same pressure tested (0.24 MPa). These treatments have shown intermediate values of NNF reductions. The third group is formed by D1, D2, D4, D7, D10, D11, D12, and D13, with the lowest NNF reduction with different pressure-time combinations. Germination showed high reduction percentages on IP_6_, raffinose, and stachyose, but an increase total phenolics, condensed tannins, and flavonoid content.

Since our investigation tried to reduce NNF content on vetches, germination may not be a suitable method to reduce all NNF, however, germination could benefit human health due to the increase of phenolic compounds. On the other hand, D3 has the lowest reduction of IP_6_ and raffinose reduction, despite having high flavonoid reduction among DIC treatments, however, IP_6_ have been demonstrated beneficial effects to human health such as reduced bioavailability of heavy metals (lead and cadmium), antioxidant activity, among others [57].

## 3. Materials and Methods

### 3.1. Seeds

Common vetch (*Vicia sativa*) was kindly provided by Campo Agropecuario Experimental of Tecnologico de Monterrey (Queretaro, Mexico). Seeds were kept under dark and dry conditions until their use.

#### Chemical Proximate Analysis

Moisture (925.10), ashes (942.05), total nitrogen (920.87), ethereal extract (945.39), and crude fiber (962.60E) were carried out in non-treated vetch flour according to AOAC official methods [58]. Results were expressed as a percentage (wet basis).

### 3.2. Vetches Treatments

Before any treatment, vetches were divided into four groups of samples: instant controlled pressure drop, cooking, germination and raw material.

#### 3.2.1. Instant Controlled Pressure Drop

An Instant Controlled Pressure Drop treatment (DIC) was carried out according to Pedrosa et al. [11] with slight modifications. One hunderd g of non-treated grounded seeds were put into the LABIC 0.1 DIC equipment (ABCAR-DIC Process, La Rochelle, France) following a central composite rotatable experimental design [59]. Saturated steam pressure (P) and thermal treatment time (t) were the independent variables. The design yielded 13 experiments with four (2^2^) factorial points, four-star points (−α, −1, 0, +1 and +α) and five central points (0,0). Pressure values ranged from 0.20 to 0.45 MPa and treatment time from 30 to 360 s. Run experimental values are shown in Table 6. After DIC treatment, obtained flours were kept under dry conditions and darkness until their use.

#### 3.2.2. Cooking Treatment

Cooking was carried out according to de Almeida Costa et al. [39] and Barampama and Simard [60] with slight modifications. First, vetch seeds were soaked in sterile water during 12 h (10% *w/v*), at 25 °C. Then, soaking water was discarded and replaced with fresh water. Seeds were cooked with boiling water (10% *w/v*) for 45, 60, 90, and 120 min, (C45, C60, C90, and C120, respectively). After cooking, seeds were dried at 45 °C until a final moisture content of 15–20% dry basis. Finally, all samples were ground and passed through 60 mesh sieve.

#### 3.2.3. Germination

Germination was carried out according to de Souza Rocha, et al. [61]. Seeds were rinsed with sterile water and kept at 25 °C under light/dark cycles (12 h). Once cotyledons sprouted about 10 cm (5 to 6 days), germinated seeds were also dried at 45 °C until a final moisture content of 15–20%. Dried samples were milled to pass 60 mesh sieves.

### 3.3. Non-Nutritional Factors Evaluation

#### 3.3.1. Methanolic Extracts Preparation

1 g of grounded seeds (treated and not treated) were extracted with 20 mL of methanol with HCl (1%) in agitation (130 rpm) during 2 h under dark conditions at 25 °C. Methanolic extracts were filtered and stored in the dark at −20 °C until analysis [62].

#### 3.3.2. Total Phenolics Quantification

Total phenolics were estimated according to Singleton et al. [63] with slight modifications. 150 µL of water was added to 20 µL of methanolic extract and oxidized with 50 µL of Folin-Ciocalteu reagent (0.5 N), then, neutralized with 50 µL of sodium carbonate solution (20% *w/v*). The mixture was incubated for 2 h at 25 °C. After the incubation time, absorbance was measured at 760 nm using an xMark Microplate Spectrophotometer (Bio-Rad, Hercules, CA, USA). A standard curve of gallic acid (0 to 300 µM) was done. Results were expressed as mg of gallic acid equivalent per g of vetch flour dry weight. Samples were analyzed in triplicates.

#### 3.3.3. Flavonoids Quantification

Quantitative determination of flavonoid content was performed by mixing 50 µl of a methanolic extract with 180 µL of distilled water and 20 µL of 2-aminoethyldiphenyl borate solution (10 g/L) [64]. After 10 min, absorbance was measured at 404 nm using a 96 micro well flat-bottom plate using an xMark Microplate Spectrophotometer (Bio-Rad, Hercules, CA, USA). Extracts absorbance was compared with a standard curve of rutin (0 to 0.05 g/mL). Flavonoid content was expressed as mg rutin equivalent per g of vetch flour dry weight. Samples were analyzed in triplicate.

#### 3.3.4. Tannins Quantification

Tannins were quantified by spectrophotometric vanillin assay [65,66] adapted to microplates. 20 µL extract were mixed with 100 µL vanillin reagent (vanillin 1% plus 8% HCl 1:1 *v/v*). The mixture was incubated at 30 °C for 30 min. Absorbance was measured at 500 nm (xMark Microplate Spectrophotometer). Results were expressed as mg equivalent of catechin (compared with the standard curve from 0.25 to 1 mg/mL) per g of vetch flour dry weight. Samples were analyzed in triplicate.

#### 3.3.5. Oligosaccharides Quantification

5 mL of ethanol (50% *v/v*) was added to 0.5 g of vetch flour and was homogenized for 1 min in Ultra-turrax (IKA Works Inc., Wilmington, DE, USA). Afterward, centrifugation (HERMLE Z383K, Wehingen, Germany) was carried out at 2 817 g (4 °C, 10 min) recovering supernatant. This process was carried out three times. The supernatants were collected and passed through a solid phase extraction (SPE) SampliQ C-18 column (200 mg bonded silica, 3 mL, 45 µm, 1200 series, Agilent Technologies, Santa Clara, CA, USA) previously activated with 3 mL distilled water and 3 mL of methanol. Oligosaccharides collected were vacuum-dried in a rotary evaporator and resuspended with 1 mL of distilled water, then filtered through 0.45 µm membrane [67]. Extracts were analyzed with a liquid chromatographer (Agilent Technologies 1200 series) with a refraction index detector. A 20 µL injection of oligosaccharides was passed through a Zorbax Carbohydrate column (4.6 × 150 mm, 3.5 µm, Agilent Technologies). The mobile phase contained acetonitrile-water (60:40 *v/v*) and set to a flow rate of 1 mL/min. The concentration was calculated using a calibration curve of raffinose and stachyose (0–2 mg/mL) [68]. Results were expressed as mg per g of vetch flour dry weight.

#### 3.3.6. Phytates Quantification

For phytates determination, 10 mL of HCl 0.5 M was added to 0.5 g of vetch flour and homogenized in Ultra-Turrax (IKA Works Inc.) during 1 min; then, mixtures were centrifugated at 2 817 g (4 °C, 10 min) recovering supernatant. This procedure was done three times. The total supernatants collected were passed through a strong anionic exchange (SAX) column (100 mg bonded silica, 1 mL, 45 µm) (Agilent Technologies 1200 series) activated with 5 mL methanol, then 5 mL HCl 0.5 M. The filtrate was discarded, and the phytates retained in column were eluted with 2 mL HCl 2 M, vacuum-dried in a rotary evaporator and resuspended with 2 mL of a solution containing 51.5 mL methanol, 48.5 mL distilled water, 1.6 mL of tert-butyl ammonium hydroxide (TBNOH) (Fluka Analytical, Charlotte, NC, USA), 0.2 mL sulfuric acid 5 M and 1 mL formic acid (91%) and filtered through a 0.45 µm membrane prior to vial incorporation. A 20 µL injection of the filtered solution was passed through a reverse phase (Zorbax Eclipse XDB C-18 column, 4.6 × 150 mm, 5 µm) for HPLC analysis using a diode array detector. The mobile phase was the same solution used to resuspend phytates, with a flow rate of 1 mL/min. The identification of phytic acid was done by comparing the retention time of sodium phytate (IP_6_) standard [69]. IP6 was expressed as phytic acid mg per g of vetch flour dry weight.

#### 3.3.7. Statistical Analysis

Statistical Analysis was performed using the Statistica software (TIBCO Software Inc., Palo Alto, CA, USA). For each treatment analysis of variance (ANOVA) and multiple comparisons by Tukey’s honest significant test (α < 0.05) was applied to evaluate any significant difference. In the case of the experimental design of DIC treatment, statistical analysis was also performed by the surface response methodology. Finally, for all the responses, principal component analysis (PCA) was also performed to visualize the distance and relatedness between treatments.

## 4. Conclusions

To improve the nutritional profiles of pulses, this study focused on the effect of Instant Controlled Pressure Drop technology (DIC), cooking and germination on the reduction of IP_6_, raffinose, stachyose, total phenolics, flavonoids and condensed tannins of vetches. By cooking, it was possible to reduce significantly the NNFs of vetches, however long thermal treatments could cause adverse effects on nutrients such as lipids degradation or vitamin loss. Germination also removed IP_6_ and oligosaccharides (raffinose and stachyose) well and, it also may lead to an increase of total phenolics, flavonoids, and condensed tannins. This could be beneficial since total phenolics, flavonoids, and condensed tannins have been demonstrated to increase some important bioactivities for human health, for instance, antioxidant activity. Moreover, according to the literature, the selected operation conditions during germination directly impact the NNFs content. Therefore, further studies are needed to evaluate the effect of the main variables during germination such as seed water content, lightness or darkness, temperature, and days of germination. The instant controlled pressure drop (DIC) method was an effective technology to reduce NNFs of vetches in only 5 min, getting similar results to cooking, which exerted the highest reductions. Further studies must be performed to evaluate the effect of the initial moisture content of seeds, the particle size, and the possibility to couple DIC process in tandem with soaking and germination, as well as other novel technologies such as ultrasound and microwaves to achieve optimal NNFs levels. Moreover DIC can be easily adapted at an industrial scale. Nevertheless, in all the cases, more studies are needed to evaluate the effect of cooking, germination and DIC treatment on antinutritional factors with protein nature, and the effect of these treatments over the bioactive compounds present in vetches.

## Figures and Tables

**Table 1:** Chemical proximate analysis of non-treated vetch flour.

Moisture (%)	15.0 ± 0.5
Ashes (%)	2.5 ± 0.1
Crude fiber (%)	12.1 ± 0.6
Total nitrogen (%)	16.1 ± 0.9
Ethereal extract (%)	5.0 ± 0.9
Nitrogen-free extract (%) ^1^	49.3

Values are expressed as means of three replicates ± SD. Calculated by difference.

**Table 2:** Total phenolics content, condensed tannins content, total flavonoids content, phytic acid content, raffinose and Stachyose content of DIC, cooked, germinated and raw vetches.

Sample	TPC ^1^ (mg/g)	CT ^2^ (mg/g)	TFC ^3^ (mg/m)	IP_6_^4^ (mg/g)	Raffinose (mg/g)	Stachyose (mg/g)
DIC1	150.25 ± 0.22	10.16 ± 0.90	12.13 ± 0.22	10.40 ± 0.62	4.32 ± 0.03	47.20 ± 1.10
DIC2	129.20 ± 0.45	9.10 ± 0.70	12.35 ± 0.45	8.48 ± 0.71	4.98 ± 0.08	38.50 ± 0.60
DIC3	112.54 ± 0.41	9.41 ± 0.90	5.68 ± 0.04	11.87 ± 0.80	6.67 ± 0.11	53.82 ± 1.40
DIC4	178.96 ± 0.46	11.54 ± 0.70	6.45 ± 0.03	10.04 ± 0.39	4.38 ± 0.07	45.73 ± 1.12
DIC5	99.53 ± 0.17	8.83 ± 0.80	11.17 ± 0.24	0.99 ± 0.11	2.25 ± 0.01	4.41 ± 0.22
DIC6	147.38 ± 0.22	9.96 ± 1.02	11.78 ± 0.14	1.02 ± 0.32	1.78 ± 0.00	4.66 ± 0.33
DIC7	98.38 ± 0.27	10.16 ± 0.81	14.38 ± 0.17	10.38 ± 1.17	4.77 ± 0.45	47.69 ± 0.77
DIC8	164.60 ± 0.22	11.43 ± 0.83	6.01 ± 0.05	5.09 ± 0.72	2.00 ± 0.04	23.05 ± 0.56
DIC9	126.13 ± 0.12	10.64 ± 1.17	5.83 ± 0.03	5.08 ± 0.47	1.89 ± 0.05	23.11 ± 0.14
DIC10	152.74 ± 0.10	10.30 ± 0.66	5.40 ± 0.07	10.41 ± 1.25	4.76 ± 0.03	47.51 ± 0.36
DIC11	161.16 ± 0.54	11.05 ± 0.87	15.11 ± 0.77	6.28 ± 0.16	4.68 ± 0.01	53.15 ± 0.14
DIC12	185.47 ± 0.45	12.79 ± 0.92	13.98 ± 0.87	11.53 ± 2.16	6.89 ± 0.44	28.26 ± 0.22
DIC13	147.76 ± 0.44	10.18 ± 0.60	5.89 ± 0.11	10.49 ± 0.43	2.92 ± 0.01	47.57 ± 0.47
C45	132.45 ± 0.13	10.67 ± 0.33	11.68 ± 1.01	1.65 ± 0.91	0.68 ± 0.02	7.54 ± 0.50
C60	115.80 ± 0.36	10.12 ± 1.81	8.06 ± 0.23	0.55 ± 0.12	0.49 ± 0.02	2.57 ± 0.07
C90	212.26 ± 0.98	10.02 ± 1.27	8.47 ± 0.11	0.59 ± 0.07	0.46 ± 0.05	2.64 ± 0.01
C120	173.22 ± 0.78	9.80 ± 1.39	8.75 ± 0.19	0.63 ± 0.08	0.31 ± 0.06	3.07 ± 0.00
Germinated	136.66 ± 0.78	11.76 ± 0.70	11.68 ± 0.53	0.57 ± 0.08	0.31 ± 0.04	2.51 ± 0.17

^1^ Total Phenolics Content; ^2^ Condensed Tannins Content; ^3^ Total Flavonoids Content and ^4^ Phytic Acid Content Values are expressed as means of three replicates. DIC = Instant Controlled Pressure Drop treatments, C = Cooking treatments.

**Table 3:** Effect of DIC over total phenolics, condensed tannins, and flavonoids.

Sample	Total Phenolics Reduction (%)	Tannins Reduction (%)	Flavonoids Reduction (%)	Phytates Reduction (%)	Raffinose Reduction (%)	Stachyose Reduction (%)
DIC1	21.5 ^cd^	17.4 ^b^	26.7 ^abc^	23.0 ^c^	45.0 ^a^	23.0 ^bc^
DIC2	32.5 ^de^	26.0 ^b^	25.4 ^abc^	37.2 ^e^	36.6 ^c^	37.2 ^d^
DIC3	41.2 ^ef^	23.5 ^b^	65.7 ^c^	12.1 ^a^	15.1 ^b^	12.2 ^a^
DIC4	5.6 ^ab^	6.2 ^ab^	61.0^abc^	25.6 ^d^	44.3 ^e^	25.4 ^c^
DIC5	48.0 ^ef^	28.2 ^b^	32.5 ^abc^	92.7 ^h^	71.4 ^g^	92.8 ^g^
DIC6	23.9 ^cd^	19.0^ab^	28.8 ^abc^	92.4 ^h^	77.4 ^i^	92.4 ^g^
DIC7	48.6 ^f^	17.4 ^b^	13.1 ^a^	23.1 ^c^	39.3 ^d^	22.2 ^b^
DIC8	14.0 ^abc^	7.1 ^ab^	63.7 ^bc^	62.3 ^g^	74.5 ^h^	62.4 ^f^
DIC9	34.1 ^de^	13.5 ^ab^	64.8 ^bc^	62.4 ^g^	75.9 ^hi^	62.3 ^f^
DIC10	20.2 ^abc^	16.3 ^ab^	67.4 ^bc^	22.9 ^c^	39.4 ^d^	22.5 ^bc^
DIC11	15.8 ^abc^	10.2 ^ab^	8.7 ^a^	53.5 ^f^	40.5 ^d^	13.3 ^a^
DIC12	3.1 ^a^	−4.0^a^	15.5 ^ab^	14.6 ^b^	12.3 ^a^	53.9 ^e^
DIC13	22.8 ^cd^	17.2 ^ab^	64.4 ^bc^	22.3 ^c^	62.9 ^f^	22.4 ^bc^

Values are expressed as means of three replicates. Letters in the same column indicate significative differences. DIC= Instant Controlled Pressure Drop treatments, C= Cooking treatments.

**Table 4:** Effect of cooking over total phenolics, condensed tannins, and flavonoids.

Sample	Total Phenolics Reduction (%)	Tannins Reduction (%)	Flavonoids Reduction (%)	Phytates Reduction (%)	Raffinose Reduction (%)	Stachyose Reduction (%)
C45	30.8 ^a^	13.2 ^a^	29.4 ^b^	87.8 ^b^	91.3 ^b^	87.7 ^b^
C60	39.5 ^a^	17.7 ^a^	51.2 ^a^	95.9 ^a^	93.8 ^a^	95.8 ^a^
C90	–10.9 ^b^	18.5 ^a^	48.8 ^a^	95.6 ^a^	94.2 ^a^	95.7 ^a^
C120	9.5 ^c^	20.3 ^a^	47.1 ^a^	95.3 ^a^	96.1 ^c^	95.0 ^c^

Values are expressed as means of three replicates. Letters in the same column indicate significative differences.

**Table 5:** Effect of germination on NNF.

NNF	Reduction (%)	Increase (%)
Total phenolics	-	28.6 ± 3.1
Flavonoids	-	27.2 ± 2.8
Condensed Tannins	-	4.4 ± 0.0
IP6	95.8 ± 0.2	-
Raffinose	96.1 ± 0.6	-
Stachyose	95.9 ± 0.0	-

Values are expressed as means of three replicates ± SD.

**Table 6:** Vetch flour DIC processing conditions.

Sample	Saturated Steam Pressure (MPa)	Treatment Time (s)
DIC1	0.33	195
DIC2	0.45	195
DIC3	0.33	360
DIC4	0.33	195
DIC5	0.41	312
DIC6	0.41	78
DIC7	0.33	195
DIC8	0.24	78
DIC9	0.24	312
DIC10	0.33	195
DIC11	0.20	195
DIC12	0.33	30
DIC13	0.33	195
